# Emergent Percutaneous Rotational Atherectomy to Bailout Surgical Transapical Aortic Valve Implantation: A Successful Case of Heart Team Turnaround

**DOI:** 10.5935/abc.20190235

**Published:** 2019-12

**Authors:** Tawfiq Choudhury, Shahrukh N. Bakar, Bob Kiaii, Patrick Teefy

**Affiliations:** 1 London Health Sciences Centre - Interventional Cardiology, London, Ontario - Canada

**Keywords:** Aortic Valve Stenosis, Atherectomy, Coronary, Atherectomy, Peripheral Arterial Disease, Coronary Angiography.

## Abstract

Transcatheter aortic valve implantation (TAVI) is an established treatment for severe aortic stenosis (AS) in patients with elevated surgical risk. Concomitant coronary artery disease affects 55-70% of patients with severe AS. Percutaneous coronary intervention in patients with TAVI can be challenging. We report a case of acute coronary obstruction immediately following transapical TAVI deployment requiring emergent rotational atherectomy.

## Introduction

Transcatheter aortic valve implantation (TAVI) is an established treatment for severe aortic stenosis (AS) in patients with elevated surgical risk. Concomitant coronary artery disease affects 55-70% of patients with severe AS.^1^ Percutaneous coronary intervention (PCI) in patients with TAVI can be challenging. Rotational atherectomy (RA) before or after TAVI has been described in an elective setting, but not as an emergent procedure.^2,3^ Coronary artery occlusion or obstruction is a rare but serious complication of TAVI. We report a case of acute coronary obstruction immediately following transapical TAVI deployment requiring emergent RA to restore adequate perfusion.

### Case Report

**Figure 1:**
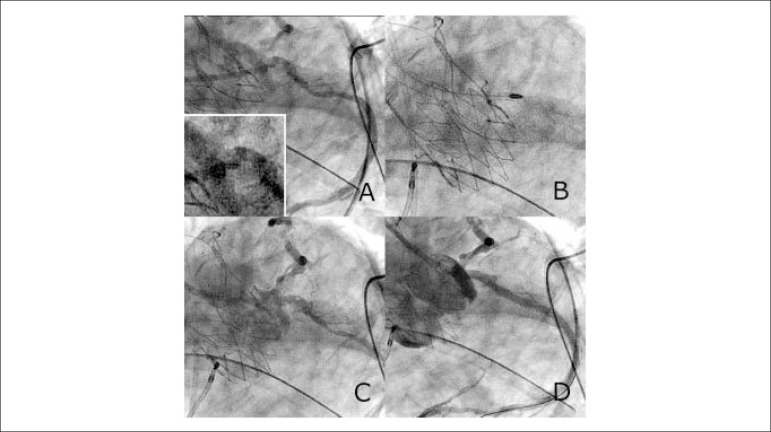
Percutaneous coronary intervention to circumflex artery lesion. A) Emergent coronary angiogram showing the new ostial left circumflex filling defect and prior mid‑circumflex lesion. Inset view shows ostial left circumflex lesion at greater magnification. B) Rotablator 1.5 mm burr entering culprit ostial left circumflex artery lesion. C) Ostial left circumflex lesion after rotational atherectomy shows angiographic improvement. D) Final angiographic result after stent insertion and high-pressure post-dilatation.

An 86-year-old male, with prior coronary artery bypass grafting and severe peripheral arterial disease (PAD), presented with New York Heart Association class III exertional dyspnea. Echocardiography revealed severe calcific AS with normal left ventricular systolic function. Cardiac computed tomography (CT) showed adequate left (14 mm) and right (21 mm) coronary heights. Previous coronary angiography had demonstrated non-occlusive triple-vessel coronary artery disease with a functional left internal mammary artery graft to the left anterior descending artery and a dominant native left circumflex artery. A Symetis Acurate ‘Large’ (Boston Scientific,

Boston, MA, USA) TAVI prosthesis was deployed transapically in the hybrid operating theatre. Immediately thereafter, the patient became hypotensive and developed posterolateral ST-segment elevation. Emergent coronary angiography showed a critical, calcific filling defect at the junction of the distal end of the short left mainstem and proximal-mid circumflex arteries (Figure 1, Panel A). Through the radial access, a 6-French Cordis XB 3.5 guide catheter (Cardinal Health, Vaughan, ON, Canada) was used to cannulate the left main coronary artery. Heparin was administered to maintain ACT > 250 seconds and clopidogrel 600mg was administered. The lesion resisted extensive attempts at balloon delivery. A 0.009” RotaWire Floppy guidewire was inserted to facilitate the 1.5 mm Rotablator Rotational Atherectomy System (Boston Scientific Corporation, Boston, MA, USA) burr passage at 180,000 rpm. Three passes were undertaken into the mid-circumflex artery (Figure 1, Panel B). A 2.5 x 20 mm non-compliant balloon was subsequently inserted unimpeded in the left main coronary artery (post-RA and balloon dilatation-figure 1, panel C) extending into the proximal circumflex segment over a Pilot 50 guidewire (Abbott Vascular, Abbott Park, IL, USA). A 3.25 x 38 mm Xience Xpedition (Abbott Vascular, Abbott Park, IL, USA) drug-eluting stent was successfully deployed extending from the ostium of the left main coronary artery into the proximal-mid circumflex lesions and post-dilated with a 3.5 x 20 mm non-compliant balloon at high pressures with a good angiographic result (Figure 1, Panel D) and resolution of electrocardiographic changes along with marked hemodynamic improvement. The patient subsequently recovered uneventfully in the intensive care unit and was extubated the following day and transferred to the ward uneventfully. Peak creatine kinase and high-sensitivity troponin T levels were 961 U/l and 1921 ng/l respectively.

## Comments

PCI post-TAVI can be challenging. The case report describes emergency RA immediately after deployment of a transapical TAVI prosthesis and highlights the feasibility and challenges of complex, high-risk PCI in such patients.

**Figure 2:**
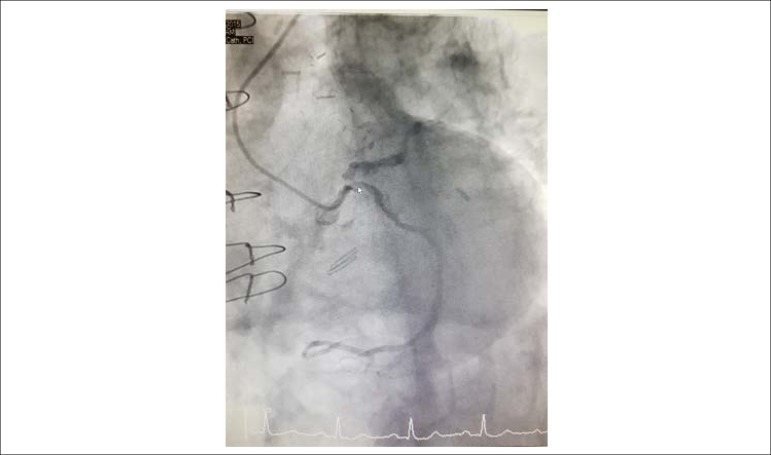
Baseline Coronary Angiogram. Baseline, pre-TAVI coronary angiogram showing calcified left coronary system, including calcified left main, ostial left circumflex and left anterior descending arteries.

**Figure 3:**
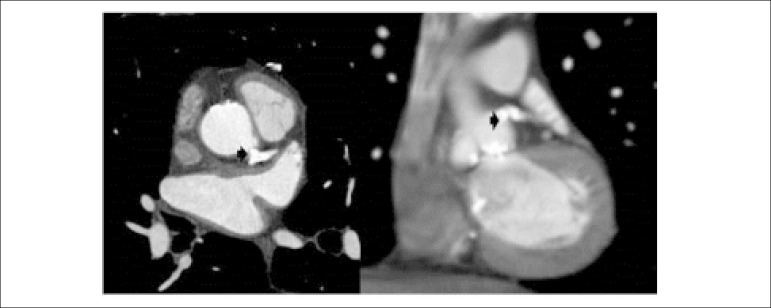
Cardiac CT. Pre-TAVI cardiac CT showing heavy calcification (arrow) extending into the left main ostium, left anterior descending artery and left circumflex artery as well as annular calcium.

**Figure 4:**
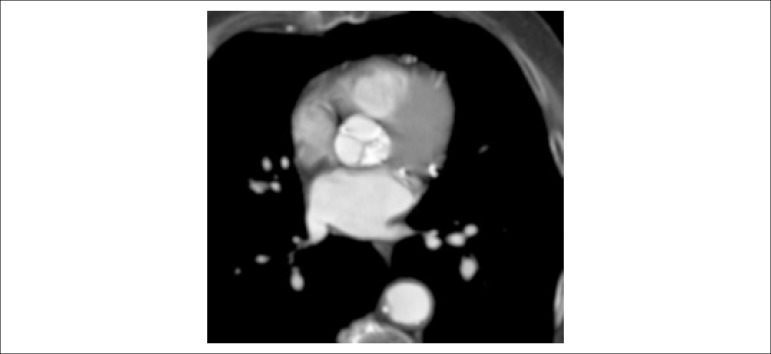
Annular calcium. Pre-TAVI cardiac CT demonstrating heavy aortic valve annular calcification.

Choice of vascular access for PCI can be limited to only transradial in patients with severe PAD. Anatomical variants and tortuosity can impede guide manipulation. The valve prosthesis can obstruct coronary ostia or alter annular geometry and a trial with multiple guides might be necessary for selective engagement. Valves jailing the coronary ostia can make selective intubation more difficult.^1^ The Symetis Acurate TA TAVI prosthesis pulls the native valve leaflets away from the coronary ostia making coronary obstruction unlikely.^4^ However, coronary flow can be compromised by displacement of annular calcium into the ostium, as in our case (Figures 2-4). Modification of coronary lesions may require RA to debulk calcific deposits permitting passage of stents and adequate expansion. The rate of major RA-related complications (in-hospital death, cardiac tamponade, and emergent surgery) was 1.3% according to a Japanese registry, increased with age and was approximately 4 times higher if RA was performed in an emergency setting of coronary artery disease per se.^5^ Previous use of RA in TAVI patients have been in an elective setting unlike our report. RA in a TAVI setting poses additional challenges, particularly with suboptimal guide engagement.

## Conclusion

This case highlights the complexity of coronary obstruction following TAVI and the need for availability of alternate arterial access (i.e. radial) and various modalities of revascularization (i.e. RA). Importantly, it highlights the necessity of a heart team approach with the seamless and unencumbered transition from a surgical domain (transapical TAVI) to the interventional realm (PCI with RA). Pre-procedural CT guided planning in terms of prosthesis selection, implantation technique, and bailout strategy in case of coronary compromise is also of critical importance.
